# An observational feasibility study on the impact of green exposure on major depressive episode symptomatology and inflammatory biomarkers

**DOI:** 10.3389/fpsyt.2025.1631393

**Published:** 2025-11-10

**Authors:** Gianna Pavarino, Claudio Brasso, Marina Boido, Anna Carluccio, Francesca Cirulli, Giulio Mengozzi, Roberta Schellino, Alessandro Vercelli, Paola Rocca

**Affiliations:** 1 Neuroscience Institute Cavalieri Ottolenghi, Orbassano, Italy; 2 Department of Neuroscience R. Levi Montalcini, University of Turin, Turin, Italy; 3 Struttura Complessa Psichiatria Universitaria, Dipartimento di Neuroscienze e Salute Mentale, Azienda Ospedaliero-Universitaria Città della Salute e della Scienza di Torino, Turin, Italy; 4 Center for Behavioral Sciences and Mental Health, Istituto Superiore di Sanità, Rome, Italy; 5 Dipartimento di Scienze Mediche, University of Turin, Turin, Italy; 6 Struttura Complessa Biochimica Clinica, Dipartimento di Medicina di Laboratorio, Azienda Ospedalie-ro-Universitaria Città della Salute e della Scienza di Torino, Turin, Italy

**Keywords:** major depressive disorder, bipolar disorder, interleukin-6, C-reactive protein, adiponectin, human-nature interaction, Shinrin-yoku, urban green environments

## Abstract

**Introduction:**

Major depressive and bipolar disorders are prevalent mental health conditions sharing the presence of major depressive episodes (MDEs). While psychopharmacological and psychological therapies are first-line treatments for MDEs, the response is often incomplete. New approaches focused on the human-nature relationship might complement antidepressant treatments, improving response.

**Methods:**

This observational pilot study aims to assess the feasibility of implementing regular exposure to green environments such as woods, forests, large parks, and gardens for at least forty-five minutes twice a week in a sample of patients experiencing a MDE who require adjustments to their antidepressant therapy. It also has the purpose of detecting changes in symptoms and inflammatory biomarkers at follow-up after six weeks.

**Results:**

Fifty-three patients were evaluated at the baseline; thirty-one completed the study. Nineteen (61%) of the completers reported regular exposure to greenery during the study. At follow-up, actively exposed patients showed trends of improvements in depressive symptoms, lower levels of C-reactive protein and interleukin-6, and higher adiponectin concentrations.

**Discussion:**

This result suggests that incorporating green exposure into clinical practice is feasible and potentially useful. However, more rigorous evaluations on larger samples are needed to verify whether exposure to greenery may complement MDEs treatment and favorably impact MDE-associated inflammatory processes.

## 1 Introduction

Mood disorders are defined as a group of psychiatric disorders characterized by the recurrence of clinically significant changes in mood state, energy, cognitive processes, sleep, or appetite (1, 2). Within this group, Major Depressive Disorder (MDD) and Bipolar Disorder (BD) are the most common conditions in the general population (1), with a prevalence of 5 and 1-2%, respectively (3). MDD is the second cause of years lived with disability and is expected to become the leading one by 2030 (4, 5); BD is currently the 27^th^ (4). Globally, both conditions are associated with significantly reduced quality of life and life expectancy (6, 7) and carry a particularly elevated risk of premature death from natural causes (8) and suicide (8–11). A common feature of both MDD and BD is the occurrence of major depressive episodes (MDEs), which consist of periods lasting at least two weeks and characterized by depressed mood, loss of interest, disturbances in sleep and appetite, fatigue, cognitive impairment, feelings of guilt, and recurrent thoughts of death (2). Therapeutic strategies, such as antidepressant medication and psychotherapy, are available to treat MDEs and are widely recommended as first-line intervention for depression (12); although outcomes are suboptimal given that roughly half of the patients do not achieve complete remission (13–16). Inadequate response to treatments represents a significant burden for patients and their families and a relevant economic and social cost due to lost working days, reduced productivity, and high utilization of healthcare services (17).

This lack of response to first-line treatments may partially depend on the complex and poorly understood etiopathogenesis of mood disorders (18). Scientific evidence suggests that during a MDE (19) patients experience a phase of emotional, cognitive, and inflammatory disruption, accompanied by acute psychological and physical stress. The central nervous system (CNS) and the rest of the body establish a complex dialogue through cytokines, metabolism, and acute-phase proteins (engaging the psycho-neuro-endocrine-immune systems) (20), creating a dysfunctional feedback loop that negatively influences mood regulation, immune response, and stress adaptation (20). Recent research on the pathophysiological processes of depression has moved the focus from the classical alterations of the monoamine neurotransmitter systems, suggesting that a complex interplay between dysregulation of the hypothalamus-pituitary-adrenal axis (HPA) and the immune system, genetic susceptibility, maladaptive epigenetic modifications, oxidative stress-induced damage, and neurodevelopmental alterations may all contribute mechanistically to pathophysiology (21–29). Focusing on plasmatic biomarkers, MDEs in MDD and BD are associated with prolonged exposure to cortisol in response to stressors (30–32), higher levels of acute phase proteins, mainly C-reactive protein (CRP) (33, 34), complement components C3 and C4 (35–40), and pro-inflammatory interleukin-6 (IL-6) (34, 41–43), and altered metabolic profiles in terms of leptin-resistance induced hyperleptinemia (44, 45) and reduced adiponectin levels (46–50). Finally, inflammation-related brain-derived neurotrophic factor (BDNF) reduced expression is linked to cognitive and affective symptoms found in MDEs (51–54).

The high rate of patients who do not adequately respond to first-line therapies motivates the development of additional nonpharmacological treatments for MDEs in MDD and BD. Among them, there is a growing interest in multidisciplinary approaches, also known as nature-based solutions, that focus on human-nature interaction, including exposure to natural green environments (55). A recent large meta-analysis demonstrated a significant correlation between living in greener areas and reduced depression risk, with a 10% increase in green space associated with a 3.7% decrease in depression odds (56). Other studies have reported that living near green spaces reduces proinflammatory processes and chronic stress response and is associated with a lower risk of developing depression (57, 58) and a longer life expectancy (59). Green exposure effectively fosters a solid human-nature connection, consistent with the so-called “biophilia hypothesis” (60). Introduced in 1984, this theory suggests that humans have an innate connection with nature, shaped by evolution, which contributes to psychological well-being by reducing stress and enhancing cognitive function (61). A concept rooted in this idea is “Shinrin-yoku” - literally forest bathing - a Japanese practice of physical relaxation through forest aerosol showers with all senses (sight, hearing, smell, and touch) (62–64), which has been shown to lower cortisol levels, pulse rate, and blood pressure, promoting a sense of well-being (65). Accordingly, the stress reduction theory (66) posits that natural environments help alleviate stress by dampening the nervous system’s reaction to stressors, while the attention restoration theory (67) suggests that time in nature replenishes cognitive resources and enhances concentration. Beyond cognitive benefits, green exposure fosters positive affect and social connectedness, vitality, and well-being (68), all protective factors in mood disorders, while promoting opportunities for social interaction, which can counteract the social withdrawal commonly observed in depression (69).

Some of these effects of frequenting green spaces may depend on the increased exposure to molecules released in green areas, i.e., terpenes - plant secondary metabolites - that display a broad spectrum of biological activities, particularly anti-inflammatory (70) and anxiolytic ones. Once inhaled, they can affect the CNS and several brain structures, by activating olfactory receptors and subsequently influencing limbic regions such as the amygdala and hippocampus, potentially helping to reduce stress and depressive symptom (71, 72), Alternatively, they may cross the BBB, where they may modulate neurotransmission involved in mood regulation (73), particularly through the binding with cannabinoid receptors 2 (CB2) (74–76) and γ-Aminobutyric acid type A (GABA-A) receptors (77–79). Other studies indicate that visual exposure to greenery alone (such as 3D images, videos, or virtual reality of green landscapes) can elicit similar benefits by activating brain areas related to emotion regulation (80, 81), consequently reducing stress and improving mood (82, 83). Additionally, sunlight exposure in green spaces may enhance antidepressant effects through vitamin D activation (84). These effects of light exposure are probably exerted through the activation of the retina, which in turn modulates the activity of different limbic, thalamic, and hypothalamic structures, including the suprachiasmatic nucleus, the ventrolateral preoptic nucleus, orexin areas, the amygdala, the nucleus accumbens, the perihabenular nucleus, the left hippocampus, the ventral lateral geniculate nucleus, the intergeniculate leaflet, and the lateral habenula (85).

Therefore, given the abovementioned positive effects of green exposure on depressive symptoms, stress, and inflammation and their basic mechanisms of action, we expect that repeated and regular exposure to natural greenery may benefit MDD and BD patients experiencing a MDE. Following this hypothesis, this observational pilot study has a twofold aim: first, to assess the feasibility of implementing regular green exposure in a clinical sample during the acute phase of illness (MDE) by examining recruitment, engagement with the intervention, and attrition rates; second, to explore whether participation in the intervention is associated with a decrease in MDE-related depressive symptoms and inflammatory biomarkers.

## 2 Materials and methods

### 2.1 Participants and study design

Participants were enrolled from May 2023 to March 2024 at the Struttura Complessa Psichiatria Universitaria, Dipartimento di Neuroscienze e Salute Mentale, Azienda Ospedaliero-Universitaria “Città della Salute e della Scienza di Torino”, Turin, Italy. Clinical assessment and venous blood sampling of all subjects were performed at this location.

The patients recruited were outpatients aged between 18 and 65 years, living in an urban area, and having a diagnosis of MDD or BD (according to the *Diagnostic and Statistical Manual of Mental Disorders, Fifth Edition, Text Revision* (DSM-5-TR) criteria) with an ongoing MDE necessitating the initiation or modification of antidepressant treatment. MDD, BD, and MDE were diagnosed according to the DSM-5-TR criteria and assessed with the Structured Clinical Interview for DSM-5-TR Research Version (SCID-RV). Exclusion criteria were neurodevelopmental disorders, alcohol and/or substance use disorders, cognitive impairment, intellectual disability, and psychiatric comorbidities (e.g., obsessive-compulsive disorders and anxiety disorders), difficulties with walking or mobility that impair access to green areas, and pregnancy.

Due to the lack of clear reference values for the biological markers analyzed, we recruited a sample of healthy volunteers (HV). HV were defined as people not affected by any mental disorder and with a negative family history for MDD and/or BD up to second-degree relatives. To minimize potential confounding factors in the comparison between patients and HV, we selected a sample of HV comparable to the clinical sample in terms of age, sex, education, and community of origin, specifically urban areas. Diagnoses of mental disorders were excluded using the same standardized clinical tools (SCID-5-RV).

The study had a prospective design: patients were assessed by a psychiatrist, and a venous blood sample was taken at baseline (t0), i.e., during the MDE at the time of antidepressant therapy modification, and after six weeks at the follow-up visit (t1).

At baseline, patients were instructed to spend time in natural green environments. We defined green environments as all those areas with low anthropization that allow patients to be in contact with nature while distancing themselves as much as possible from the urban environment. Therefore, we considered green environments to be forests, woodlands, and extensive urban gardens or parks, allowing patients an immersive greenery experience through sight, hearing, and smell. This operational definition aligns with the Japanese concept of Shinrin-Yoku (62, 63, 65). We suggested patients be exposed to green environments for 45–60 minutes, three non-consecutive days per week, until the follow-up visit. Exposure was explained as simply spending time in nature without engaging in physical activity. Since no established scientific protocols recommended a specific duration of green exposure, we based our approach on the WHO Guidelines on Physical Activity and Sedentary Behavior, which advise at least 45 minutes of physical activity three times a week on non-consecutive days (86). Following this model, we similarly suggested a comparable frequency and duration for green exposure. HV’s data collection and blood sampling were only performed at one time point, since the only information regarding green exposure that was collected for this group concerned their usual habits, namely whether or not they had frequented green environments during the six weeks prior to recruitment.

Comprehensive information was provided on the procedures and goals of the study, and signed consent was obtained from all subjects. The study was designed in accordance with the Declaration of Helsinki and was approved by the Local Research Ethics Committee (Protocol number: 0010765).

### 2.2 Exposure to green, clinical, and biological markers assessment

At the baseline, all participants were evaluated using a semi-structured interview to assess socio-demographic characteristics and to investigate the relationship with green in terms of time spent in green environments, including parks, gardens, and woods, in the six weeks before the interview. HV were interviewed to assess whether they met the exclusion criteria and were considered in the exposed to greenery group if they stayed in green environments for at least 45 minutes on two non-consecutive days per week for the six weeks before enrollment and blood sampling, otherwise they were considered in the non-exposed to greenery group.

The clinical characteristics of MDD or BD and the severity of depressive symptoms were assessed by two expert psychiatrists (C.B.; A.C.) The severity of symptoms was evaluated with the Hamilton Rating Scale for Depression (HAM-D) (87), where a higher score indicates greater severity.

After six weeks from baseline (t0), patients performed the follow-up visit (t1). They were reassessed with the HAM-D scale and asked whether, during the six weeks of the study, they were exposed to green environments for at least 45 minutes on two non-consecutive days per week. According to their response at the six-week follow-up visit (t1), patients were classified as exposed to or nonexposed to green environments during the study, i.e., between t0 and t1. This classification was applied to divide patients into groups both at follow-up (t1) and retrospectively at baseline (t0).

The nursing staff conducted venous sampling of all subjects at baseline and follow-up. In the serum, we measured the concentration of cortisol, CRP, IL-6, C3 and C4 complement fractions, leptin, and adiponectin at the Struttura Complessa Biochimica Clinica, Azienda Ospedaliero-Universitaria “Città della Salute e della Scienza”, Turin, Italy, and BDNF at “Neuroscience Institute Cavalieri Ottolenghi” laboratory in Orbassano.

The procedure for measuring biomarker concentrations in participants’ blood samples is described in the **Supplementary Material**.

### 2.3 Statistical analysis

The normal distribution of the continuous variables was verified with the Shapiro-Wilk test. Since the sample size was relatively small, to provide a more robust and appropriate summary of the data, variables were expressed as median and interquartile range.

The Mann-Whitney test was employed for independent sample comparisons, and the Wilcoxon test for paired samples. The effect size of the comparisons was calculated using the rank biserial correlation method. The results were reported as absolute values. The false discovery rate (FDR) was controlled with the Benjamini-Hochberg (BH) correction procedure and set at.05 (88).

The significant results of the comparisons between exposed and nonexposed patients at follow-up (t1) and within exposed or nonexposed patients between baseline (t0) and follow-up (t1) were used to perform linear backward regression models. Follow-up (t1) variables showing a significant variation from the baseline in the between- and/or within-group comparisons were chosen as outcomes for the regression models. Exposure to green environments between baseline and follow-up was entered as the regressor into all regression models. The models were controlled for baseline (t0) diagnosis (MDD or BD), depressive symptoms, drug naivety, age, sex, education, body mass index (BMI), self-reported physical activity, and relationship with green, and for the baseline (t0) variable corresponding to the dependent follow-up (t1) variable. Regarding this latter control, for example, if IL-6 at follow-up (t1) was the outcome of the regression model, we controlled for IL-6 at baseline (t1) as an additional control variable. The regressor and control variables entered the regression model as independent variables.

We set the alpha error at .05, the beta error at .10, and the effect size for non-parametric comparisons at .5. All comparisons were one-tailed, assuming a reduction in depressive symptoms and inflammatory biomarkers concentrations in participants who reported engaging in green exposure. Independent comparison had a between-groups ratio of 1.5. For multiple linear regressions, we set the alpha error at .05, the beta error at .10, three significant control predictors, and an effect size f^2^ of .15 related to the reported participation in the intervention of green exposure. This led to a minimal sample size of 38 patients for paired comparisons, 58 and 88 for independent ones, and 73 for multiple linear regressions.

Analyses were conducted with the IBM Statistical Package for Social Science (SPSS), version 29.0; Jasp, version 0.18.3; and Gpower, version 3.1.

## 3 Results

### 3.1 Feasibility of the intervention

**Figure 1:**
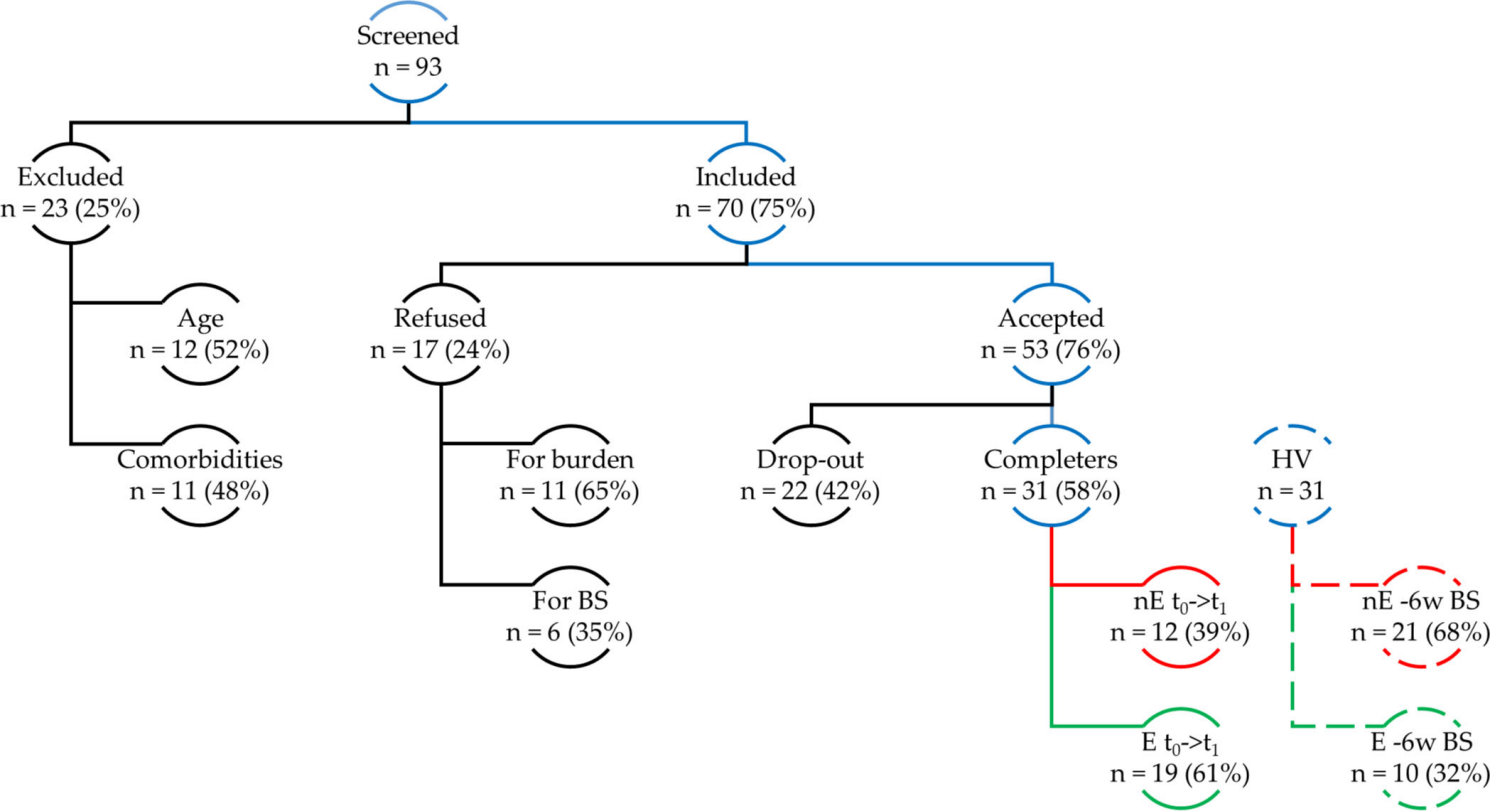
Flowchart of participants. A total of 93 patients were screened; 23 were excluded, and 70 passed screening. Among these, 17 declined participation. Of the 53 enrolled, 22 dropped out after baseline, leaving 31 completers: 12 nonexposed (nE) and 19 exposed (E) to greenery ≥40 min twice weekly. Additionally, 31 healthy volunteers (HV) participated: 21 nE and 10 E based on self-reported green exposure in the 6 weeks before blood sampling. Exposure was voluntary and self-reported. BS: blood sampling; HV: healthy volunteers; nE: nonexposed; E: exposed; t0→t1: baseline to six-week follow-up; –6w BS: six weeks before sampling.

Ninety-three patients during a MDE requiring a modification of the antidepressant were screened. Twenty-three (24.7%) were excluded for age or mental and/or physical comorbidities, constituting an exclusion criterion. Of the seventy eligible patients, seventeen (24.3%) refused to participate in the study because they felt it was burdensome or did not want to take additional blood samples. Fifty-three patients were recruited at baseline; twenty-two participants dropped out at follow-up, and 31 (58.5%) completed the study, leading to a drop-out rate of 41.5%. Nineteen completers (61%) engaged with the intervention, reporting regular exposure to greenery during the six weeks of the study observation. Consequently, the attrition rate for the suggested intervention was 39%. These results are represented in **Figure 1**.

### 3.2 *Post-hoc* statistical power and considerations on inferential analyses

All inferential analyses were underpowered. In particular, the *post-hoc* statistical power of the pairwise comparison was 65% for exposed patients and 47% for nonexposed. For the between-group comparison between exposed and nonexposed patients, it was 36%, and for the linear regression, it was 55%. Due to the low statistical power, the results of the following inferential analyses will be considered as trends since the low sample size of this pilot study may not bring out significant results or, conversely, despite FDR correction, make differences evident due to small sample size “instability”.

### 3.3 Characteristics of the sample

Eighty-four subjects (53 patients, 31 HV) participated in the study. No trends suggest differences at baseline between completers and drop-outs (**Table 1**). Most completer patients were female (71%), had a median age of 47, and had an education of 13 years. The median duration of illness was six years, and the median severity of the MDE was moderate (89).

**Table 1:** Characteristics of follow-up (n = 31) and drop-out (n = 22) patients at baseline and be-tween-group comparison.

Groups of variables	Total sample of patients at t0 (n=53)	Completers patients at t0 (n=31)	Drop-out patients (n=22)	Completers *vs.* drop-out patients at t0	
				Effect size	p-FDR
Sociodemographic					
Age, years	48.0 [31.0; 56.0]	47.0 [35.0; 59.5]	49.5 [30.3; 55.0]	.012	.848
Sex, female	36 (68%)	22 (71%)	14 (64%)	–	.464
Education, years	13.0 [13.0; 17.0]	13.0 [13.0; 16.0]	13.0 [11.5; 16.0]	.162	.424
Clinical					
Duration of illness, years	6 [2; 14]	5 [2; 13]	8 [3; 14]	.016	.718
Numbers of hospitalization	1 [1; 3]	1 [1; 2]	1 [1; 3]	.258	.411
HAM-D	18 [13; 20]	17 [12; 20]	18 [16; 21]	.177	.424
Biological markers					
CRP, mg/L	0.9 [0.4; 2.1]	0.9 [0.4; 2.5]	0.8 [0.4; 1.6]	.138	.810
IL-6, pg/mL	2.0 [1.0; 4.0]	2.0 [1.0; 4.5]	2.0 [1.0; 2.0]	.275	.411
C3, g/L	1.18 [1.02; 1.41]	1.18 [1.02; 1.39]	1.19 [1.04; 1.41]	.005	.898
C4, g/L	0.29 [0.23; 0.35]	0.26 [0.21; 0.31]	0.32 [0.28; 0.41]	.379	.150
Cortisol, mcg/L	109 [79; 153]	105 [79; 127]	136 [92; 158]	.212	.424
Leptin, pg/L	10,423 [5,751; 24,111]	14,202 [5,641; 23,196]	9,349 [7,124; 22,372]	.007	.999
Adiponectin, ng/L	8,575 [6,568; 12,303]	9,087 [6,969; 13,283]	7,367 [5,999; 11,246]	.225	.411
BDNF, pg/mL	461.7 [305.5; 823.9]	402.5 [273.2; 755.5]	635.4 [427.4; 912.1]	.236	.411

Continuous variables are expressed as medians and interquartile ranges [Q1; Q3]; *vs*: versus; FDR, False Discovery Rate correction; HAM-D, Hamilton scale for Depression; CRP, C-Reactive Protein; IL, Interleukin; C3, Complement fraction 3; C4, Complement fraction 4; BDNF, Brain-Derived Neurotrophic Factor.

Sex-dependent distributions of completers and HVs’ variables are reported in **Supplementary Material** (**Supplementary Figures 1**, **2**, **Tables 1**, **2**). No trends were found after FDR correction.

**Table 2:** Characteristics at baseline and six-week follow-up of green-exposed and nonexposed patients, and between-group and paired-sample comparisons.

Groups of variables	PT_E_t0 (n=19)	PT_nE_t0 (n=12)	PT_E_t1 (n=19)	PT_nE_t1 (n=12)	PT_E_t0 *vs.* PT_nE_t0		PT_E_t1 *vs.* PT_nE_t1		PT_E t0 *vs.* t1		PT_nE t0 *vs.* t1	
					Effect size	p-FDR	Effect size	p-FDR	Effect size	p-FDR	Effect size	p-FDR
Sociodemographic												
Age, years	46.0 [28.0; 50.0]	54.5 [38.0; 60.5]	–	–	.262	.324	–	–	–	–	–	–
Sex, female	14 (74%)	8 (67%)	–	–		.839	–	–	–	–	–	–
Education, years	13.0 [13.0; 17.0]	13.0 [11.7; 13.7]	–	–	.008	.324	–	–	–	–	–	–
Biological markers												
CRP, mg/L	0.4 [0.3; 0.8]	2.5 [1.4; 4.2]	0.6 [0.4; 1.2]	3.2 [1.3; 6.2]	.796	**.002**	.681	**.005**	.235	.542	.103	.906
IL-6, pg/mL	2.00 [1.00; 3.5]	4.00 [2.8; 16.8]	0.05 [0.01; 1.00]	2.00 [1.00; 3.00]	.415	**.048**	.535	**.005**	1	**.002**	1	.081
C3, g/L	1.04 [0.96; 1.15]	1.42 [1.31; 1.53]	1.08 [1.00; 1.17]	1.38 [1.35; 1.39]	.862	**.002**	.758	**.002**	.248	.542	.077	.906
C4, g/L	0.23 [0.18; 0.28]	0.31 [0.26; 0.43]	0.23 [0.18; 0.27]	0.32 [0.22; 0.36]	.619	**.021**	.435	.095	.095	.777	.606	.290
Cortisol, mcg/L	105 [79; 129]	110 [71; 125]	136 [96; 168]	97 [92; 133]	.050	.855	.423	.070	.484	.121	.033	.906
Leptin, pg/L	6,095 [3,1; 13,1]	24,444 [22,2; 40,0]	7,099 [4,9; 9,5]	24,506 [17,6; 36,2]	.877	**.002**	.831	**.002**	.189	.551	.407	.290
Adiponectin, ng/L	12,182 [8,6; 13,9]	7,309 [5,5; 13,5]	13,599 [10,7; 15,6]	6,968 [5,2; 10,5]	.546	**.026**	.677	**.003**	.695	**.018**	.165	.853
BDNF, pg/mL	396.7 [268.3; 591.2]	416.8 [327.3; 1,032.4]	535.0 [278.9; 1,058.7]	772.6 [516.7; 1,026.4]	.046	.750	.112	.372	.562	.110	.407	.367
Depressive symptoms												
HAM-D, total score	16.0 [12.0; 19.5]	18.5 [11.5; 20.0]	9.0 [5.0; 14.5]	13.5 [8.7; 16.5]	.081	.855	.415	.188	1	**.002**	.641	.855

Numbers in bold showed a trend in the comparisons; continuous variables are expressed as medians and interquartile ranges [Q1; Q3]; *vs*: versus; PT_E: patients exposed to green between baseline and follow-up; PT_nE: patients nonexposed to green between baseline and follow-up; t0: baseline; t1: six-weeks follow-up; Exposition to greenery at least 45’ twice a week is voluntary and self-reported. FDR, False Discovery Rate correction; CRP, C-Reactive Protein; IL, Interleukin; C3, Complement fraction 3; C4, Complement fraction 4; BDNF, Brain-Derived Neurotrophic Factor; HAM-D, Hamilton scale for Depression.

### 3.4 Group comparisons of patients at baseline and follow-up

The number of patients with MDD or BD diagnosis remained stable throughout the study, with no patients experiencing a switch to hypomania or mania.

**Figure 2:**
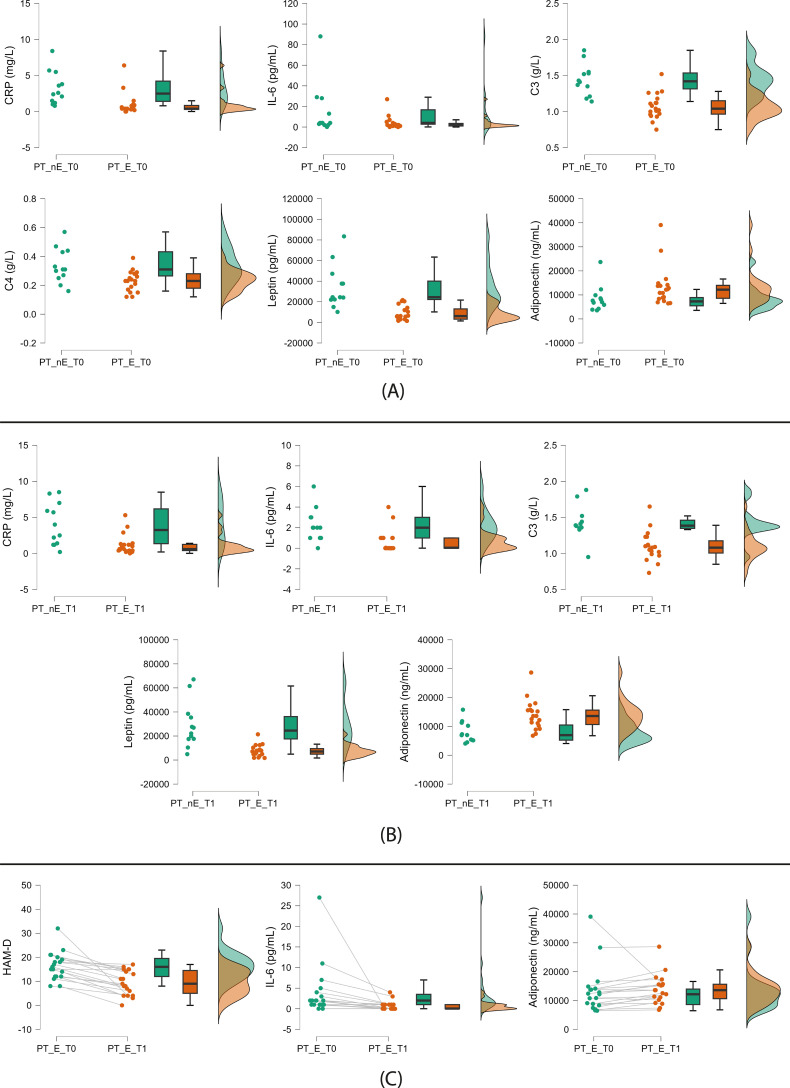
Significant trends from patient comparisons. **(A)** Nonexposed (left) vs green-exposed (right) patients at baseline; **(B)** Nonexposed (left) vs green-exposed (right) patients at follow-up; **(C)** Exposed patients at baseline (left) vs follow-up (right) (paired comparison). PT: patients; nE: nonexposed; E: exposed; T0: baseline; T1: follow-up. Green exposure refers to the six-week period between visits and is voluntary and self-reported (≥45 min twice weekly). Raincloud plots display, from left to right, dot plots (raw data), box plots (median and interquartile range), and density plots (probability distribution and estimated density).

At baseline, compared to nonexposed patients, the 19 participants who were exposed to greenery during the 6-week study follow-up showed the following trends: lower CRP, IL-6, C3, C4, and leptin levels and higher adiponectin concentrations (**Figure 2A**, **Table 2**).

At follow-up, we found the same trends except for C4 (**Figure 2B**, **Table 2**).

### 3.5 Paired sample comparisons within exposed and nonexposed patients’ groups

In the paired sample comparison of patients exposed to greenery, we found the following trends at the six-week follow-up visit: a reduction of depressive symptoms and IL-6 levels and an increase in adiponectin serum concentration (**Figure 2C**, **Table 2**).

No trends in biomarkers and depressive symptoms were found in nonexposed patients (**Table 2**).

### 3.6 Group comparisons between patients and HV at baseline and follow-up

**Figure 3:**
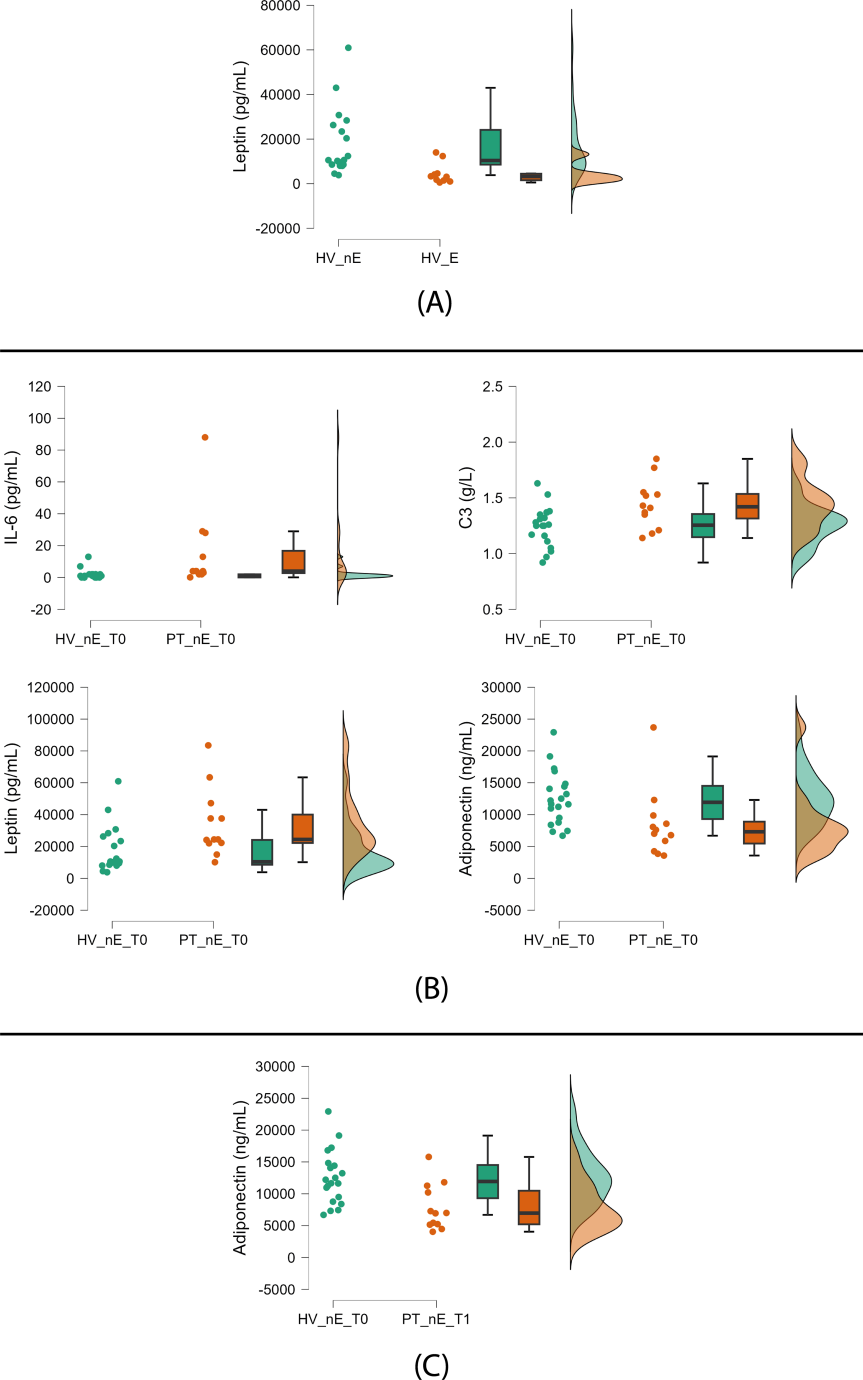
Significant trends between green-exposed and nonexposed HV and patients. **(A)** Nonexposed (left) vs green-exposed (right) healthy volunteers (HV); **(B)** Nonexposed HV (left) vs nonexposed patients (PT) (right) at baseline; **(C)** Nonexposed HV (left) vs nonexposed PT (right) at follow-up. HV: healthy volunteers; PT: patients; nE: nonexposed; E: exposed; T0: baseline; T1: follow-up. Green exposure (≥45 min twice weekly) is voluntary and self-reported. Raincloud plots display, from left to right, dot plots (raw data), box plots (median and interquartile range), and density plots (probability distribution and estimated density).

The 10 HV exposed to green in the six weeks before blood sampling (32%) had lower leptin levels than the 21 nonexposed (68%) (**Figure 3A**, **Table 3**). The comparison between patients and HV exposed to green did not show trends either at baseline or at follow-up (**Table 3**).

**Table 3:** Characteristics at baseline and six-week follow-up of green-exposed and nonexposed HV, and between-group comparisons between patients and HV.

Groups of variables	HV_E n=10)	HV_nE (n=21)	HV_E *vs.* HV_nE		HV_E *vs.* PT_E_t0		HV_E *vs.* PT_E_t1		HV_nE *vs.* PT_nE_t0		HV_nE *vs.* PT_nE_t1	
			Effect size	p-FDR	Effect size	p-FDR	Effect size	p-FDR	Effect size	p-FDR	Effect size	p-FDR
Sociodemographic												
Age, years	31.0 [25.0; 33.0]	40.5 [31.0; 57.0]	.355	.215	-.537	.243	–	–	-.260	.072	–	–
Sex, female	6 (60%)	15 (71%)	–	.832	–	.966	–	–	–	.812	–	–
Education, years	18.0 [13.0; 18.0]	13.0 [13.0; 18.0]	.177	.577	.488	.628	–	–	.198	**.048**	–	–
Biological markers												
CRP, mg/L	0.5 [0.3; 1.8]	0.9 [0.8; 1.5]	.336	.324	<.001	.656	-.082	1.00	.185	.065	.180	.094
IL-6, pg/mL	0.60 [0.01; 1.60]	1.00 [0.01; 1.75]	.273	.577	-.653	.243	-.209	.966	.440	**.004**	.073	.177
C3, g/L	1.09 [1.02; 1.11]	1.28 [1.16; 1.36]	.618	.075	.058	.735	-.02	1.00	.150	**.048**	.083	.052
C4, g/L	0.20 [0.18; 0.24]	0.27 [0.22; 0.33]	.527	.215	-.438	.735	-.336	1.00	.032	.228	.210	.431
Cortisol, mcg/L	125 [112; 144]	100 [93; 112]	-.055	.710	.124	.628	-.018	.966	.090	.530	.173	.356
Leptin, pg/L	3,192 [1,5; 4,5]	9,925 [8,6; 26,3]	.684	**.004**	-.488	.243	-.455	.248	.047	**.017**	.095	.094
Adiponectin, ng/L	13,141 [10,21; 17,29]	11,626 [9,3; 14,9]	-.177	.722	.306	.628	.018	1.00	.295	**.017**	.151	**.016**
BDNF, pg/mL	429.1 [227.4; 712.0]	234.8 [192.5; 561.7]	.1	.950	-.273	.735	-.473	.966	.085	.567	.280	.177

Numbers in bold showed a trend in the comparisons; continuous variables are expressed as medians and interquartile ranges [Q1; Q3]; *vs*: versus; HV_E: healthy volunteers exposed to green for the six weeks before blood drawing; HV_nE: healthy volunteers nonexposed to green for the six weeks before blood drawing; PT_E: patients exposed to green between baseline and follow-up; PT_nE: patients nonexposed to green between baseline and follow-up; t0: baseline; t1: six-weeks follow-up. Exposure to greenery for at least 45 minutes twice a week is voluntary and self-reported. FDR, False Discovery Rate correction; CRP, C-Reactive Protein; IL, Interleukin; C3, Complement fraction 3; C4, Complement fraction 4; BDNF, Brain-Derived Neurotrophic Factor; HAM-D, Hamilton scale for Depression.

At baseline, nonexposed patients compared to nonexposed HV showed the following trends: higher levels of IL-6, C3, and leptin and lower levels of adiponectin (**Figure 3B**, **Table 3**).

At follow-up, we found a trend of lower adiponectin serum concentrations in nonexposed patients compared to nonexposed HV (**Figure 3C**, **Table 3**).

### 3.7 The impact of green exposure on depressive symptoms severity and CRP, IL-6, and adiponectin plasmatic levels

The regression models (**Table 4**) showed the following trends of associations: patients’ adherence to the intervention of regular exposure to green environments during the six weeks of the study and a reduction of depressive symptoms, CRP and IL-6 plasmatic levels, and an increase in adiponectin plasma concentrations. No trends related to sex differences were observed (**Supplementary Material**, **Tables 1**, **2**, **Figures 1**, **2**).

**Table 4:** Regression models.

Baseline predictors	β	95%CI of β	St. β	VIF	p	Follow-up outcomes	Regression models characteristics				
							F	p	R^2^	Adj. R^2^	Durbin- Watson
Diagnosis (MDD or BD)	-3.599	-7.804;.606	-.265	1.135	.090	Depressive symptoms at t1 (HAM-D, total score)	6.019	**.001**	.481	.401	1.692
Drug naivety	-5.847	-11.132; -.563	-.345	1.152	**.031**						
Depressive symptoms at t0 (HAM-D, total score)	.461	.150;.772	.450	1.092	**.005**						
Green exposure (between t0-t1)	-3.390	-7.008;.228	-.291	1.140	**.045**						
Drug naivety	1.632	-.206; 3.471	.222	1.174	.080	CRP at t1, mg/L	13.375	**<.001**	.673	.623	2.140
Physical activity at t0	1.679	.364; 2.995	.339	1.325	**.014**						
CRP at t0	.873	.531; 1.215	.741	1.584	**<.001**						
Green exposure (between t0-t1)	-1.808	-3.232; -.385	-.357	1.486	**.015**						
Green exposure (between t0-t1)	-1.507	-2.494; -.520	-.502	1.000	**.004**	IL-6 at t1, pg/mL	9.748	**.004**	.252	.226	1.617
Adiponectin at t0	.457	.279;.635	.625	1.119	**<.001**	Adiponectin at t1, ng/L	25.578	**<.001**	.646	.621	2.222
Green exposure (between t0-t1)	3739.310	1074.1; 6404.5	.342	1.119	**.008**						

Numbers in bold showed a trend in the regressions. Exposure to greenery for at least 45 minutes twice a week is voluntary and self-reported. CI, confidence interval; St., standard; VIF, variance inflation factor; Adj., adjusted; MDD, major depressive disorder; BD, bipolar disorder; HAM-D, Hamilton scale for Depression; CRP, C-Reactive Protein; IL, Interleukin; t0, baseline; t1, follow-up.

## 4 Discussion

About 60% of completers reported adherence to the proposed intervention of regular active exposure to green environments at least twice a week during a six-week follow-up. Attrition to green exposure was relatively high (39%) but in line with previous naturalistic studies on antidepressant treatments (90). This result suggests that incorporating green exposure into clinical practice is feasible. Indeed, despite experiencing a MDE, more than half of the patients adhered to the protocol, engaging in green exposure at least twice a week for a minimum of 45 minutes. This level of adherence indicates that even individuals with significant depressive symptoms can integrate structured nature exposure into their routines, supporting its potential as a viable complementary intervention. However, it should be noted that out of 93 patients screened, only 19 (20%) reported correctly adhering to the proposed intervention (**Figure 1**). This finding limits the generalizability of the results because of several consecutive selection biases. First, exclusively screening patients with MDEs and longitudinal diagnosis of MDD or BD excludes the possibility of generalizing the feasibility of this intervention in other mental disorders or in euthymic subjects to prevent possible relapses. Second, the exclusion of patients with comorbidities and those over 65 years old does not allow the feasibility to be extended to these subject groups. Third, refusal to participate because of study-related burden or unwillingness to perform additional blood draws selected patients who were more motivated to adhere to the recommendations in the study. Fourth, there is a high dropout rate at follow-up (42%). It seems likely that dropout was largely due to logistical issues because many patients decided to continue psychiatric follow-up at services closer to their home, as returning to the hospital for follow-up visits would be inconvenient. Therefore, dropouts likely stemmed more from logistical challenges than reluctance to engage in green exposure. This may have introduced a further selection bias since only the most motivated subjects or those domiciled near the hospital completed the study. Finally, selection bias related to attrition has to be mentioned. Indeed, participants self-selected for green exposure as more motivated individuals were more likely to engage with nature.

As a second aim, we investigated whether engagement with the intervention of exposure to greenery was associated with a trend of reduction in MDE-related depressive symptoms and inflammatory biomarkers. We found trends related to symptom improvement, CRP and IL-6 reduction, and adiponectin increase. These preliminary results align with the biophilia hypothesis and previous evidence supporting the beneficial effects of nature exposure on mental health (60–63, 65–69, 91) through stress reduction, attention restoration, promotion of social interactions, and increase of vitality (66–69). In addition, we must note that our patients lived in urban areas, constantly exposed to stress-inducing stimuli typical of highly anthropized environments. Numerous studies have indeed shown that urban living is a significant risk factor for mental health disorders, including depression (92–96). Green exposure might have created a restorative experience, offering a reprieve from stressors and reducing cognitive and emotional strain while promoting relaxation and physiological balance. Regarding inflammation, we observed a trend of reduced CRP and IL-6 levels in patients who reported adherence to green exposure during the six-week follow-up. These two plasma proteins are markers of inflammatory response in various conditions, including chronic illnesses such as mood disorders. In MDD and BD, an increase in their plasma concentration is associated with ongoing MDEs and the severity of depressive symptoms (43, 97–102). We hypothesized that the observed reduction trends might partially be related to terpenes inhalation and sunlight exposure during green exposure. This putative explanation is consistent with previous studies that have reported a systemic anti-inflammatory activity of terpenes mainly through CB2 and GABA-A receptors (103–105). Also, sunlight exposure has already demonstrated an anti-depressant and IL-6-reducing effect in patients experiencing a MDE (106), probably exerted through retinal and vitamin D activation, as previously proven by other studies (84, 85). Regarding adiponectin, no previous studies have investigated its levels in patients with MDE following green exposure. However, it is well established that antidepressant treatment can modulate adiponectin concentrations in MDD and BD (49, 107–109), and that physical activity increases its levels in people with obesity (110–112) and diabetes (113). In our sample, the trend toward higher adiponectin levels among patients exposed to green environments may reflect the anti-inflammatory effects of nature exposure, potentially facilitating the restoration of adiponectin’s regulatory metabolic functions. Alternatively, patients may have increased their daily walking to reach green areas. Although analyses were adjusted for self-reported physical activity, residual confounding cannot be excluded, as actual movement levels may have been underestimated. In addition to the two main aims of the study, we found trends suggesting an inverse association between specific biological markers and adherence to the intervention of green exposure. Indeed, compared to nonexposed patients, those who actively adhere to the proposed intervention by spending more time in green environments showed a trend of lower baseline levels of CRP, IL-6, C3, C4, and leptin and higher baseline levels of adiponectin. These trends might suggest that higher inflammatory levels at baseline could be linked to lower engagement in the intervention of green exposure. Increased inflammatory markers, particularly IL-6, have been associated with specific symptoms of depression, such as hypersomnia, fatigue, leaden paralysis, and hyperphagia, which could have negatively affected patients’ adherence to the proposed intervention, specifically by reducing their ability and motivation to move toward natural green environments (114–116).

These preliminary results, although still partial and to be confirmed by further studies, suggest initial clinical relevance, indicating the potential usefulness of incorporating exposure to natural greenery as an additional treatment for depression and chronic subthreshold inflammation associated with MDEs. To implement this type of intervention in clinical practice, it would be necessary, on the one hand, to incorporate large gardens within psychiatric facilities for inpatients, and, on the other hand, to include exposure to natural greenery among the recommendations provided to outpatients experiencing a MDE (117, 118). Regarding the first type of intervention, some studies have demonstrated the effectiveness of incorporating specific green spaces into psychiatric facilities for hospitalized patients. However, these spaces are not always used or adequately designed due to organizational or financial problems within the healthcare service (118). As to the prescription of exposure to green spaces as an adjunctive therapy, patients appear to respond positively to recommendations from clinicians, provided that a good patient-clinician relationship has been established (119, 120). On the contrary, there is still some reluctance among clinicians to prescribe this type of intervention, as well as difficulties in accurately prescribing it (119, 121).

### 4.1 Limitations and strengths of the study

This study has some limitations that must be acknowledged. The sample size is highly limited, leading to low statistical power and consequent non-generalizability of the results and the impossibility of establishing sound associations, but only possible trends to be verified by future studies. The observational design of this study lacks the control capacity of a randomized trial, which may introduce biases, particularly the selection bias related to attrition. Five other sources of potential selection bias are discussed above and represent a further limitation for the generalizability of the feasibility of the intervention of active exposure to greenery proposed in this study. We focused exclusively on exposure to large green spaces, but due to the small sample size, we could not account for differences between environments, such as forests or large urban parks. Patients self-selected into green exposure, potentially leading to the exclusion of less motivated individuals. We did not use a checklist to ensure that the intervention of exposure to green environments was adequately explained to patients. Green exposure was not objectively verified, but self-reported because, for ethical and privacy reasons, using GPS tracking was impossible. This introduced uncertainty. We omitted a dose-response analysis since, at the six-week follow-up, we only had a dichotomous variable indicating whether patients frequented green spaces at least twice a week during the six weeks of follow-up. Patients were treated with different antidepressants or mood stabilizers, and we could not account for all possible variations in medication type and dosage; rather, we could only account for whether patients had a medication adjustment or a new prescription. Again, regarding possible confounding variables, the study lacks an assessment of economic status factors related to lifestyle, e.g., daily hours of sleep. Concerning regression models, we could not control for changes in psychopharmacological therapy nor socioeconomic status. However, it is worth noting that, since antidepressant therapy was set or modified at baseline, psychiatrists generally avoided major changes during the following six weeks to properly assess treatment efficacy, considering the delayed onset of antidepressant effects. This waiting period coincided with the follow-up visit of this study, likely reducing the confounding effect of therapy adjustments. Regarding the lack of assessment of socioeconomic status, we further acknowledge that socioeconomic disadvantage may have negatively impacted patients’ clinical outcomes, acting as a putative confounding variable in the analysis. Finally, concerning physical activity, we controlled for self-reported physical activity in the regression models; however, we could not exclude the possibility of inaccuracies in patients’ reporting. Therefore, part of the observed trends might be due to increased exercise rather than exposure to nature alone. The short follow-up period hinders the ability to assess long-term effects on symptom remission, functioning, and quality of life and account for the influence of seasonal variations on both mood and biomarker responses. Lastly, biomarkers in HV were measured at a single time point and, unlike patients for whom we assessed exposure to greenery during the study, this variable was assessed retrospectively, introducing possible biases.

Despite these limitations, this study has notable strengths. Unlike previous studies, which typically involve non-clinical populations, do not account for the specific phase of the illness, or focus on living in proximity to green spaces rather than exposure into them (56), this study is pioneering as the first to examine green space exposure in a clinical population (MDD and BD patients), during a specific phase of these disorders (MDE), in a real-world setting, showing that the majority of patients adhere to spending time in green spaces despite acute depression. We also show preliminary trends of improvements in depressive symptoms and inflammatory balance linked to self-reported adherence to an intervention of green space exposure. Additionally, the study’s holistic approach considers not only depressive symptoms but also factors related to inflammation, stress, metabolism, and neurotrophic factors and focuses on the human-nature interaction in terms of active exposure into green environments.

### 4.2 Future perspectives

Following the preliminary results of this study, future research should aim first at performing a randomized controlled trial on this topic, to allow stronger causal inferences and reduce selection biases. Additionally, increasing the sample size to reach higher statistical power and extending the follow-up period to eight months would help assess the long-term impacts of green exposure on patients’ functioning and quality of life, strengthening the sustainability of the benefits resulting from green exposure for a prolonged period. Furthermore, with a longer follow-up, it will also be possible to better highlight the long-term impact of green exposure, to stratify patients based on sex, on the type of green space they were exposed to, distinguishing, for example, between a dense forest or an urban park, and it will also be possible to collect more detailed data concerning frequency and duration of green exposure. Moreover, to provide more objective measures of green exposure and its immediate effects on patients' well-being, thereby reducing potential biases, it would be valuable to organize rehabilitation groups, led by a mental health expert, for example, a psychiatric rehabilitation technician, or by an expert in Shinrin-Yoku practice. By doing so, we could also consider using a checklist to assess the intervention’s explanation better and perform a dose-response analysis. Moreover, evaluating neurocognitive functions and their relationships with MDE, inflammation, and exposure to green spaces could offer insights into the underlying mechanisms. Studying epigenetic processes, such as miRNA transcription and histone modifications, could help clarify the role of green space exposure in regulating gene expression associated with depression and inflammation, further bridging the gap between environmental exposure and biological mechanisms. Finally, this protocol could be extended to anxiety and chronic stress disorders to explore the potential benefits of green exposure in these conditions as well.

### 4.3 Conclusion

In conclusion, the intervention of exposure to green environments was feasible for patients during a MDE, as more than half of the participants who attended the follow-up incorporated green exposure into their routine. This suggests that a larger study with a more rigorous evaluation might be possible in the future. Furthermore, even if underpowered due to the low number of participants completing the study and engaging in green exposure, the trends resulting from this pilot study suggest that self-reported exposure to green environments was associated with reductions in depressive symptoms, IL-6 and CRP levels, and an increase in adiponectin after six weeks. Future studies with larger sample sizes should verify the potential therapeutic benefits of green space exposure on MDEs. Information from this work is relevant to psychiatrists treating depression and epidemiologists or public mental health experts who would like to test the hypotheses of this pilot study. If the results are confirmed in larger studies, they can inform policymakers and urban green planners and consequently encourage the design and distribution of large parks throughout the cities to enhance urban mental health.

## Data Availability

The raw data supporting the conclusions of this article will be made available by the authors, without undue reservation.
