# Supplementary material for: An observational feasibility study on the impact of green exposure on major depressive episode symptomatology and inflammatory biomarkers
Source: Front Psychiatry. 2025 Nov 10;16:1631393. doi: 10.3389/fpsyt.2025.1631393 (PMC12641439; doi:10.3389/fpsyt.2025.1631393)
Supplement: Supplementary file 1 [file Supplementaryfile1.docx]

***Supplementary Material***

1. **Supplementary Methods**

Biomarkers’ dosage

BDNF was measured by sandwich ELISA (#DY248, DuoSet ELISA, R&D Systems, Minneapolis, MN, USA). The kit has a sensitivity of 23.4 pg/mL and a range of 23.4-1500 pg/mL.

IL-6 was measured by sandwich ELISA (#A16369, Access IL-6, Beckman Coulter, Brea, CA, USA). The kit has a sensitivity of 0.5 pg/mL in a 110 µL sample size and a range of 0.5-1500 pg/mL. The intra- and inter-assay coefficients of variations were 2.5% and 4.6%, respectively.

Cortisol was measured by chemiluminescent microparticle immunoassay (CMIA) (#08P3320, Alinity i, Abbott Laboratories, Sligo, Ireland). The kit has a sensitivity of 1.0 µg/dL in a 70 µL sample size and a range of 1.0-59.8 µg/dL. The intra- and inter-assay coefficients of variations were 2.5% and 4.3%, respectively.

CRP was measured by immunoturbidimetric assay (#OSR6199, Beckman Coulter AU, Ireland). The kit has a sensitivity of 0.08 mg/L in a 3 µL sample size and a range of 0.08-80 mg/L. The intra- and inter-assay coefficients of variations were 1.3% and 3.0%, respectively.

PCT was measured by CMIA (#01R1822, Alinity i B∙R∙A∙H∙M∙S PCT, Abbott Laboratories, Sligo, Ireland). The assay has a sensitivity of 0.02 ng/mL with a sample size of 150 µL and a measurement range of 0.02-100 ng/mL. The intra- and inter-assay coefficients of variation were 2.0% and 3.7%, respectively.

C3 was measured by immunoturbidimetric assay (#OSR6159, AU series, Beckman Coulter, Brea, CA, USA). The assay has a sensitivity of 0.0006 g/L in a sample size of 10 µL and a measurement range of 0.15-5.00 g/L. The intra- and inter-assay coefficients of variation were 0.76% and 0.90%, respectively.

C4 was measured by immunoturbidimetric assay (#OSR6160, AU series, Beckman Coulter, Brea, CA, USA). The assay has a sensitivity of 0.001 g/L with a sample size of 10 µL and a measurement range of 0.08-1.50 g/L. The intra- and inter-assay coefficients of variation were 1.16% and 2.52%, respectively.

Leptin was measured by sandwich ELISA (BioVendor, Brno, Czech Republic). The kit has a sensitivity of 200 pg/mL in a 100 mL sample size and a range of 1000-50,000 pg/mL. The intra- and inter-assay coefficients of variations were 4.2% and 6.7%, respectively.

Adiponectin was measured by sandwich ELISA (BioVendor, Brno, Czech Republic). The kit has a sensitivity of 470 ng/mL in a 100 mL sample size and a range of 5,000-150,000 ng/mL. The intra- and inter-assay coefficients of variations were 4.1% and 6.9%, respectively. All samples were diluted 1/300.

1. **Supplementary Figures and Tables**


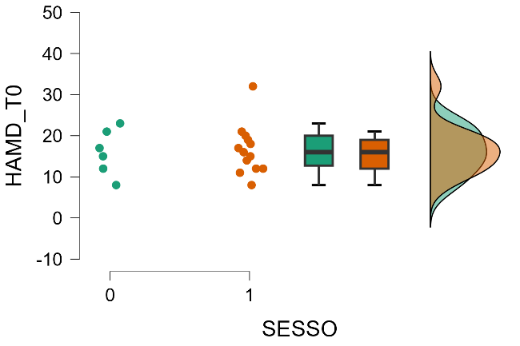

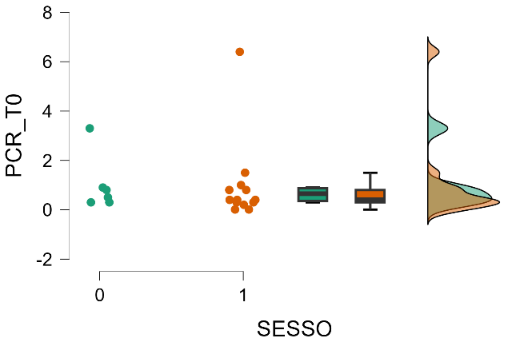

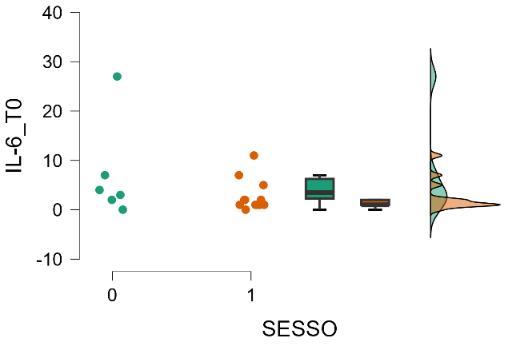


M F

M F

M F

CRP_T0


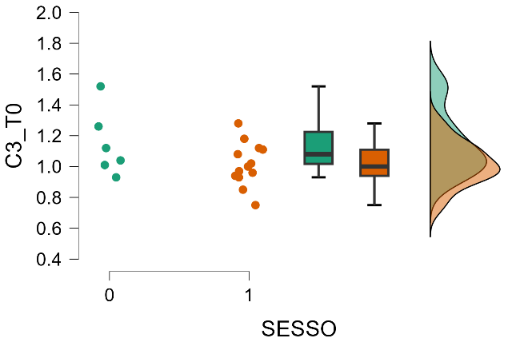

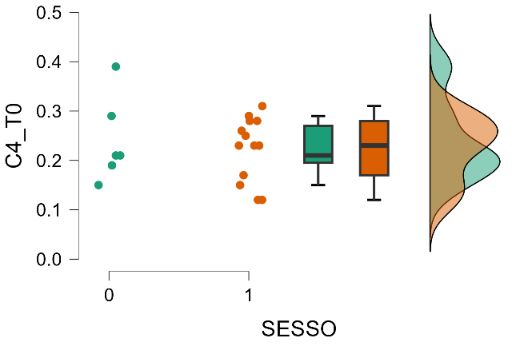

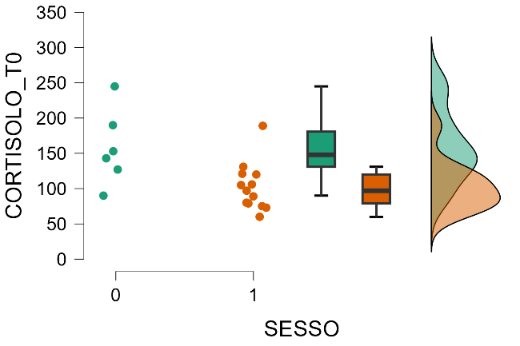


CORTISOL_T0

M F

M F

M F


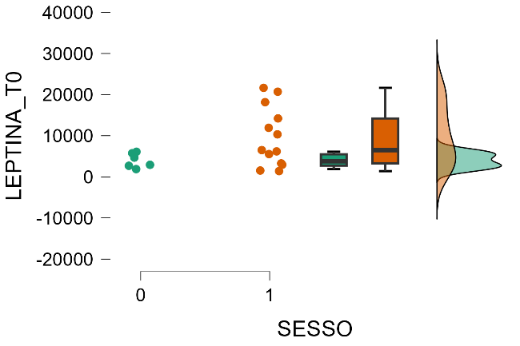

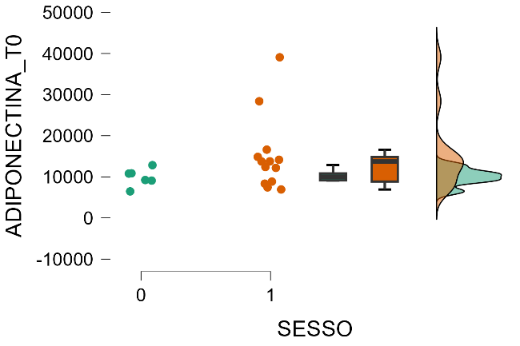

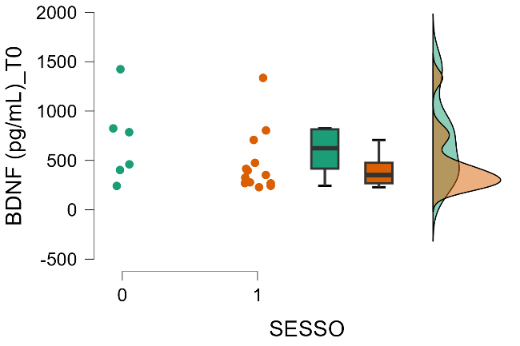


BDNF_T0

ADIPONECTIN_T0

LEPTIN_T0

M F

M F

M F

(**A**)

_______________________________________________________________________________________________________


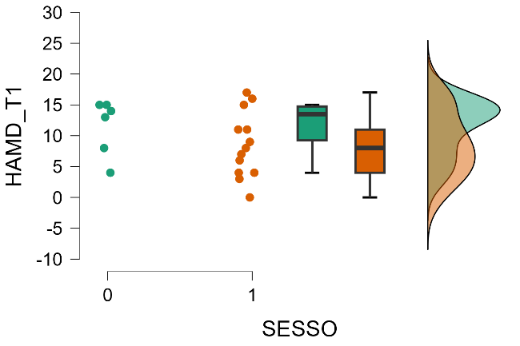

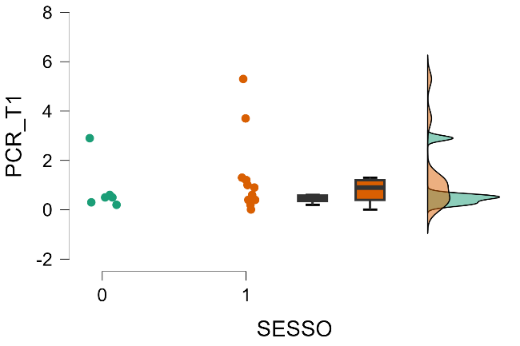

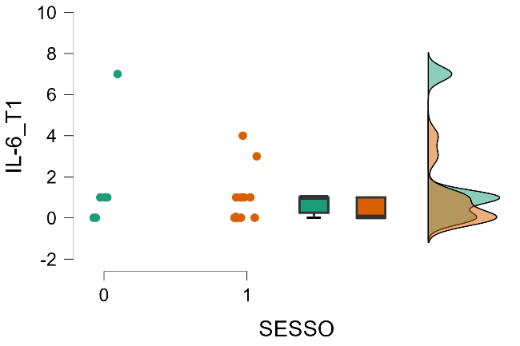


CRP_T1

M F

M F

M F


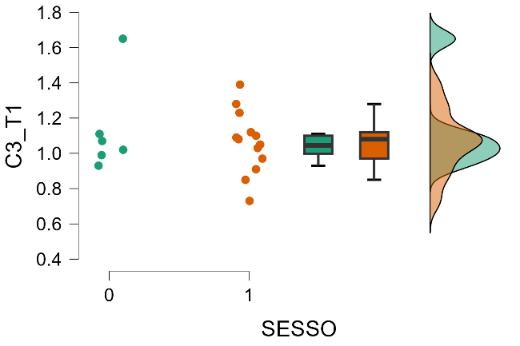

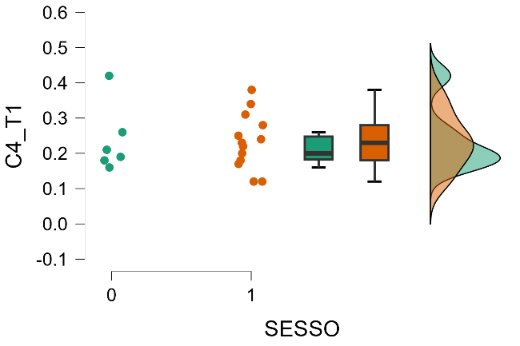

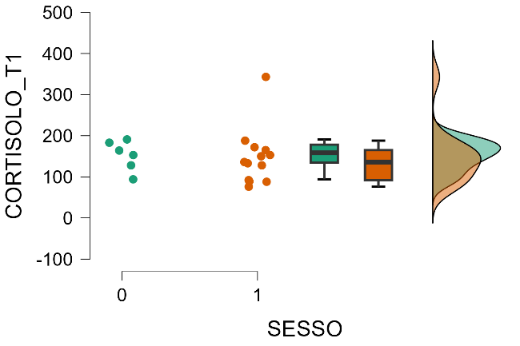


CORTISOL_T1

M F

M F

M F


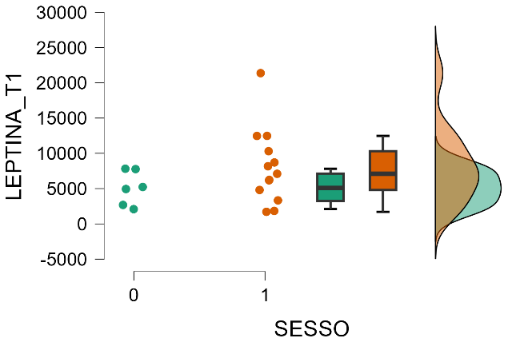

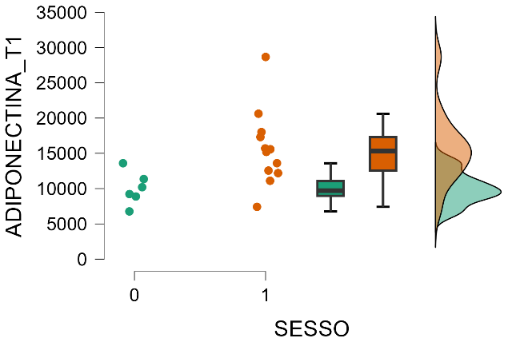

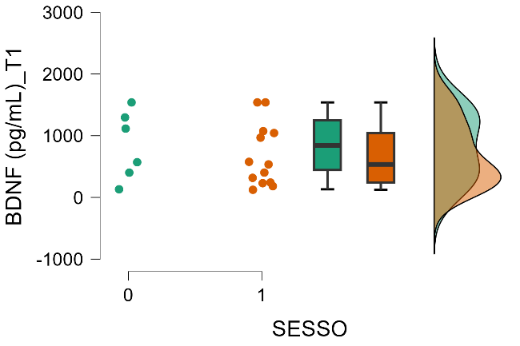


BDNF_T1

ADIPONECTIN_T1

LEPTIN_T1

M F

M F

M F

(**B**)

_______________________________________________________________________________________________________


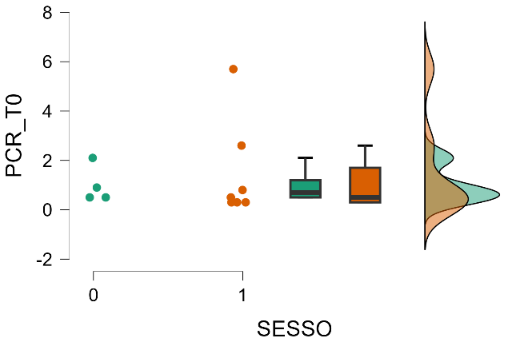

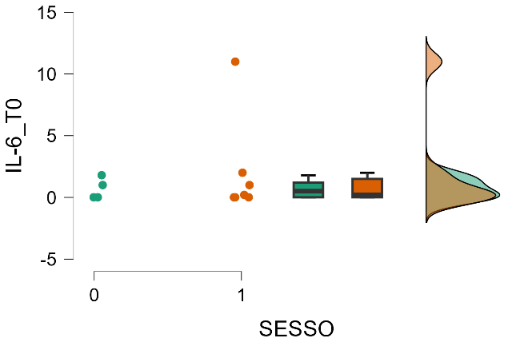


CRP_T0

M F

M F


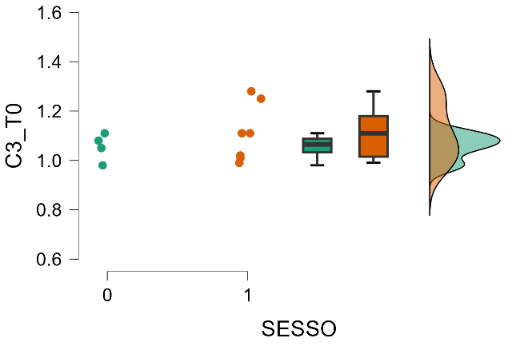

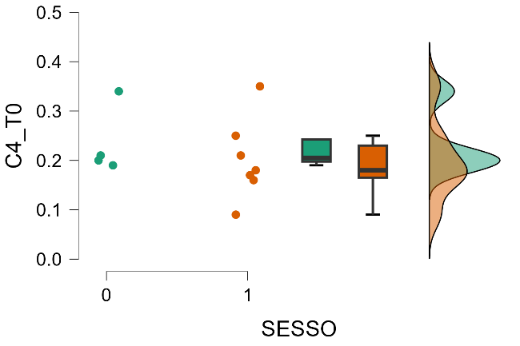

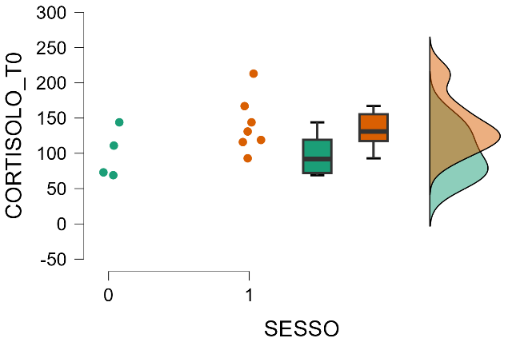


CORTISOL_T0

M F

M F

M F


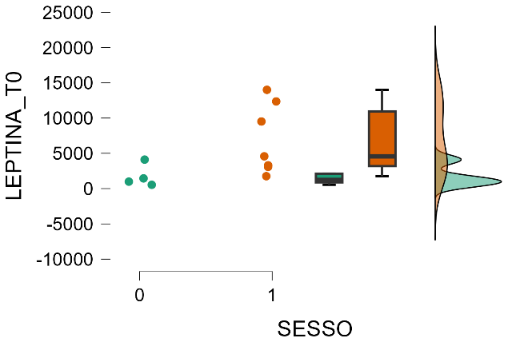

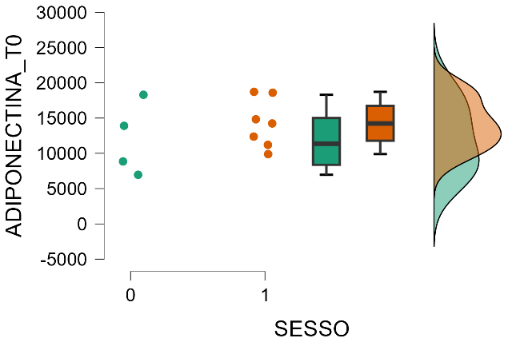

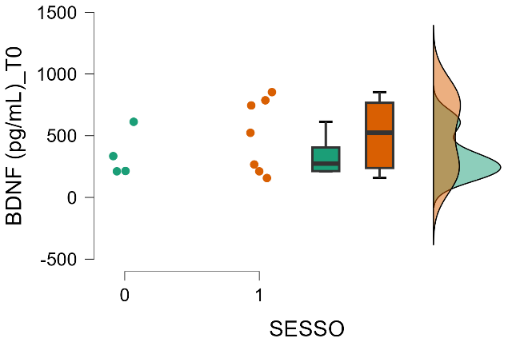


BDNF_T0

ADIPONECTIN_T0

LEPTIN_T0

M F

M F

M F

(**C**)

**Supplementary Figure 1.** Sex-dependent distributions of variables of (**A**) completer patients exposed to green at baseline (t0); (**B**) completer patients exposed to green at follow-up (t1); (**C**) HVs exposed to green. M: male; F: female.

**Supplementary Table 1**. Sex-dependent distributions of variables of completer patients at baseline (t0) and follow-up (t1), and HVs exposed to green.

| **Groups of variables** | **Patients at t0** | | **Patients at t1** | | **HV** | | **Male**  **vs. female**  **patients**  **at t0** | **Male**  **vs. female**  **patients**  **at t1** | **Male**  **vs. female**  **HV** |
| --- | --- | --- | --- | --- | --- | --- | --- | --- | --- |
|  | **Male**  **(n=6)** | **Female**  **(n=14)** | **Male**  **(n=6)** | **Female**  **(n=14)** | **Male**  **(n=4)** | **Female**  **(n=7)** | **p** | **p** | **p** |
| **Clinical** |  |  |  |  |  |  |  |  |  |
| HAM-D | 16.00 (5.59) | 16.64 (5.79) | 11.5 (4.51) | 9.0 (5.36) | - | - | 1.0 | .455 | - |
|  |  |  |  |  |  |  |  |  |  |
| **Biological markers** |  |  |  |  |  |  |  |  |  |
| CRP, mg/L | 1.02 (1.15) | 0.94 (1.62) | 0.83 (1.02) | 1.29 (1.46) | 1.00 (0.76) | 1.50 (2.03) | .533 | .385 | .630 |
| IL-6, pg/mL | 7.17 (9.99) | 2.64 (3.03) | 1.67 (2.66) | 0.79 (1.25) | 0.71 (0.87) | 2.03 (4.02) | .191 | .407 | .842 |
| C3, g/L | 1.15 (0.21) | 1.03 (0.15) | 1.13 (0.26) | 1.08 (0.18) | 1.06 (0.06) | 1.11 (0.12) | .322 | .902 | .504 |
| C4, g/L | 0.24 (0.09) | 0.23 (0.06) | 0.24 (0.10) | 0.23 (0.08) | 0.24 (0.07) | 0.20 (0.08) | .967 | .804 | .449 |
| Cortisol, mcg/L | 158.00 (53.71) | 99.86 (33.33) | 152.17 (36.24) | 143.64 (67.32) | 99.25 (35.33) | 140.43 (39.54) | .012 | .343 | .130 |
| Leptin, pg/L | 4002.00 (1747.48) | 10342.29 (7509.44) | 5086.83 (2427.41) | 8421.50 (5309.92) | 1770.25 (1603.37) | 6945.00 (4942.01) | .076 | .207 | .042 |
| Adiponectin, ng/L | 9879.50 (2156.77) | 14520.93 (8995.95) | 10003.67 (2330.33) | 15175.79 (5234.85) | 12004.00 (5123.606) | 14265.72 (3442.20) | .239 | .015 | .315 |
| BDNF, pg/mL | 689.55 (424.77) | 451.57 (309.23) | 842.38 (554.99) | 653.34 (490.38) | 343.31 (188.54) | 506.33 (295.21) | .153 | .433 | .648 |

Continuous variables are expressed as mean and standard deviation (SE); vs: versus; HAM-D: Hamilton scale for Depression; CRP: C-Reactive Protein; IL: Interleukin; C3: Complement fraction 3; C4: Complement fraction 4; BDNF: Brain-Derived Neurotrophic Factor.


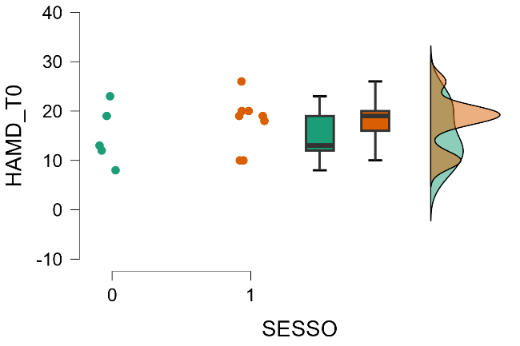

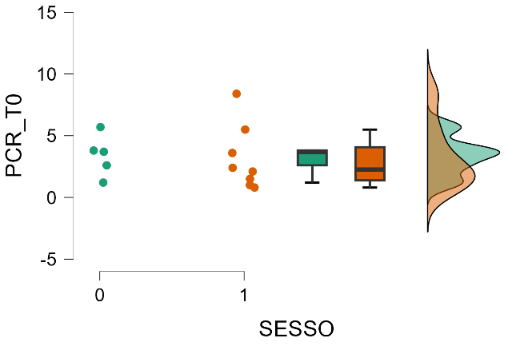

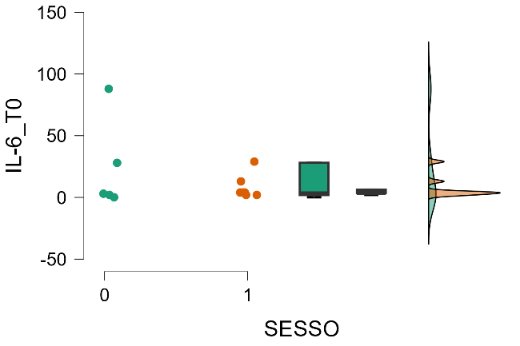


M F

M F

M F

CRP_T0


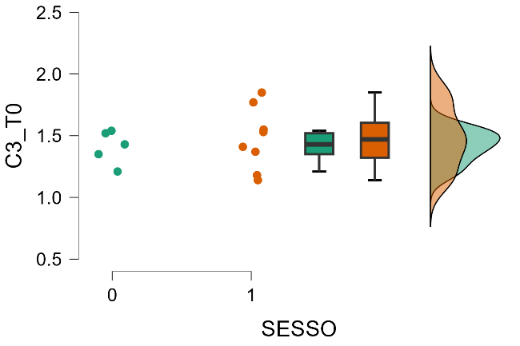

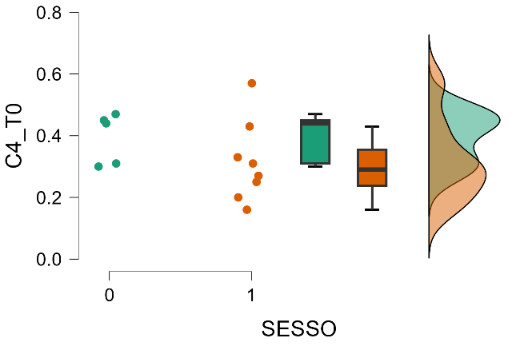

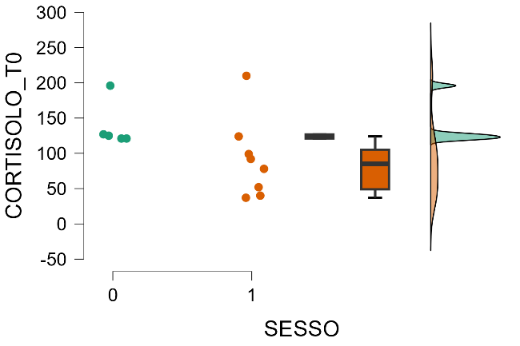


CORTISOL_T0

M F

M F

M F


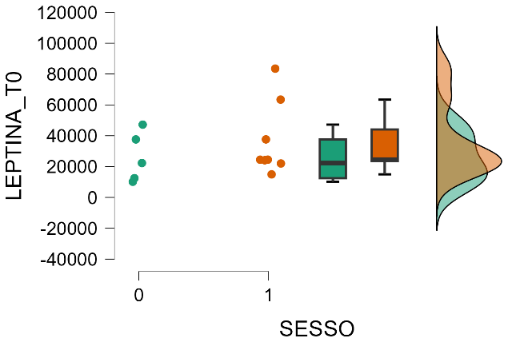

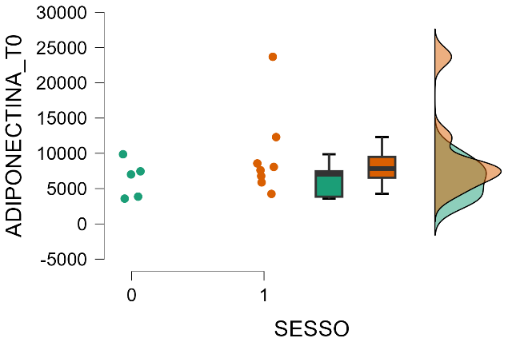

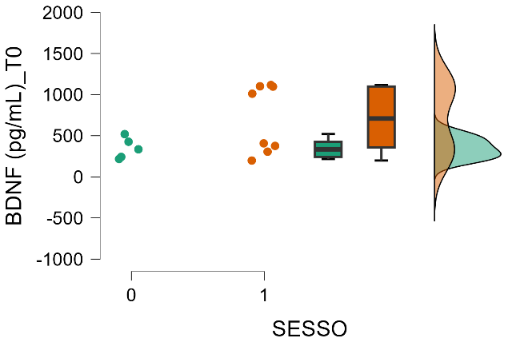


BDNF_T0

ADIPONECTIN_T0

LEPTIN_T0

M F

M F

M F

(**A**)

_______________________________________________________________________________________________________


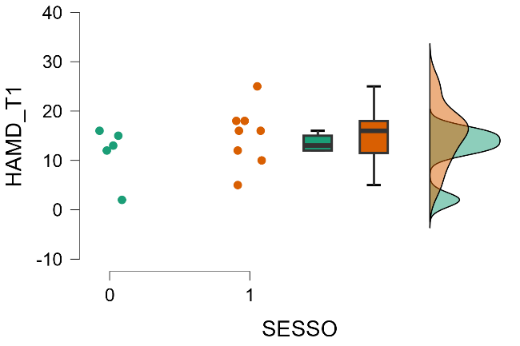

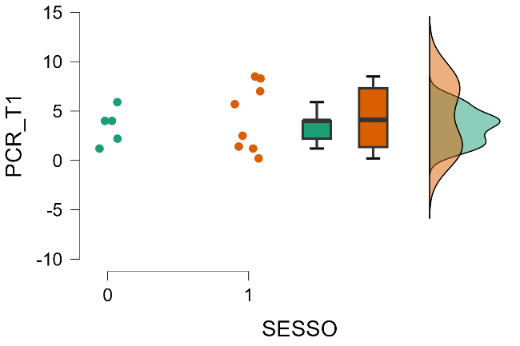

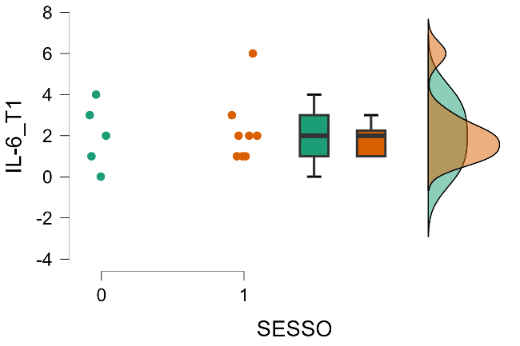


CRP_T1

M F

M F

M F


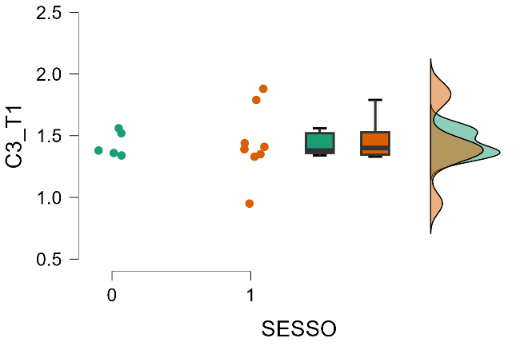

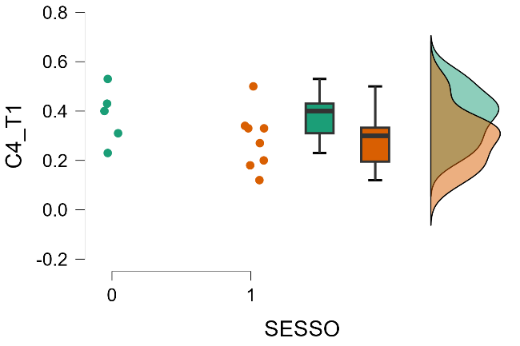

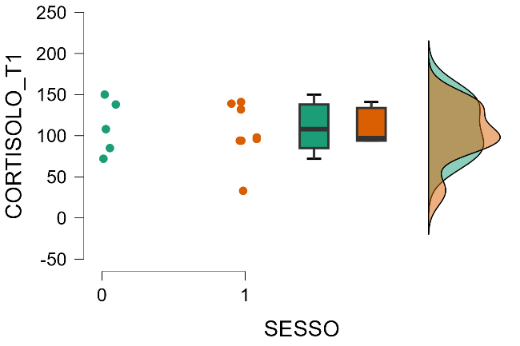


CORTISOL_T1

M F

M F

M F


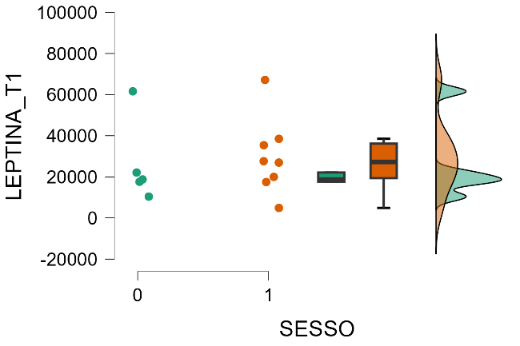

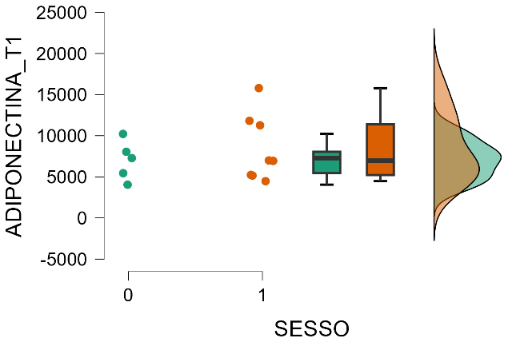

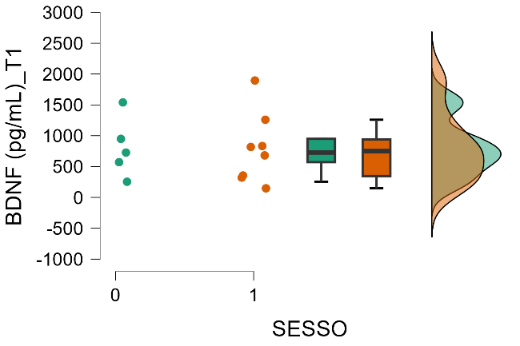


BDNF_T1

ADIPONECTIN_T1

LEPTIN_T1

M F

M F

M F

(**B**)

_______________________________________________________________________________________________________


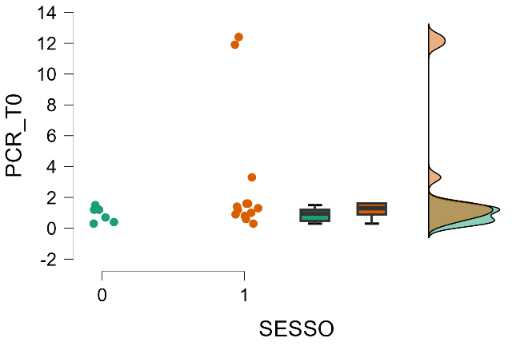

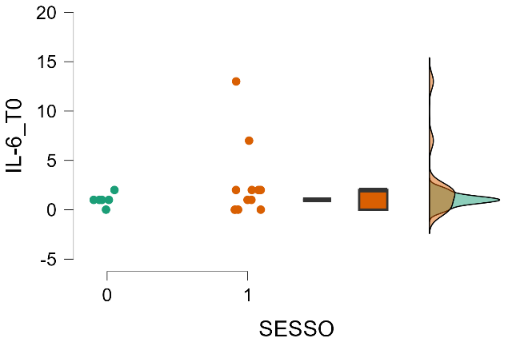


CRP_T0

M F

M F


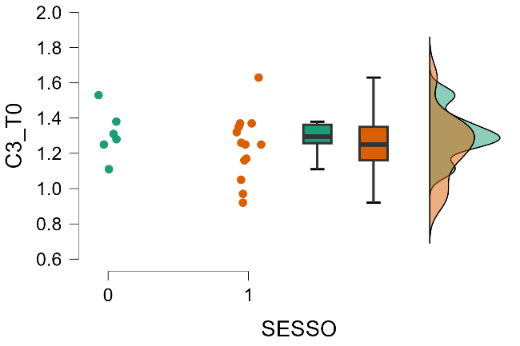

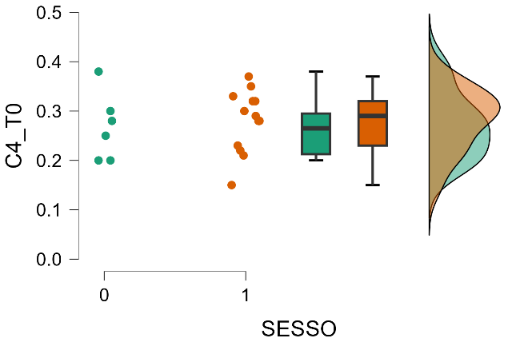

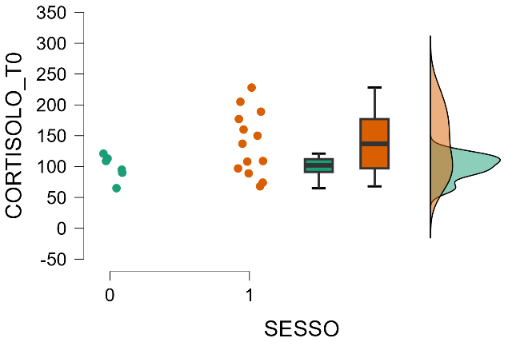


CORTISOL_T0

M F

M F

M F


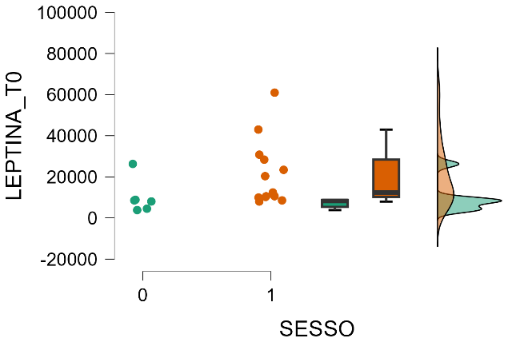

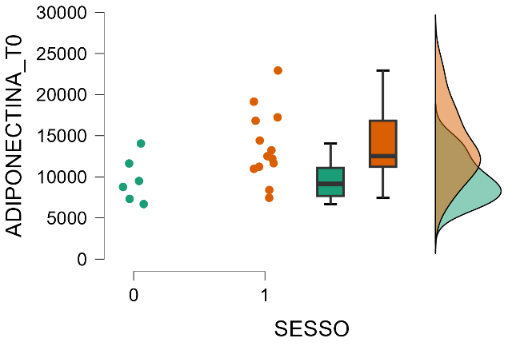

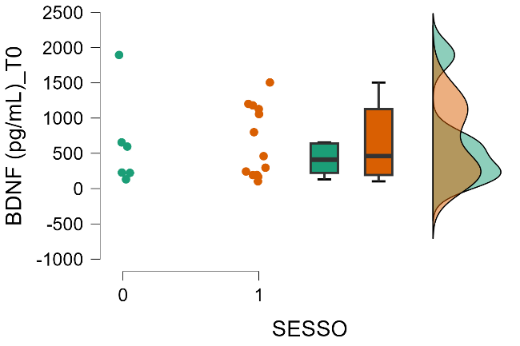


BDNF_T0

ADIPONECTIN_T0

LEPTIN_T0

M F

M F

M F

(**C**)

**Supplementary Figure 2.** Sex-dependent distributions of variables of (**A**) completer patients nonexposed to green at baseline (t0); (**B**) completer patients nonexposed to green at follow-up (t1); (**C**) HVs nonexposed to green. M: male; F: female.

**Supplementary Table 2**. Sex-dependent distributions of variables of completer patients at baseline (t0) and follow-up (t1), and HVs nonexposed to green.

| **Groups of variables** | **Patients at t0** | | **Patients at t1** | | **HV** | | **Male**  **vs. female**  **patients**  **at t0** | **Male**  **vs. female**  **patients**  **at t1** | **Male**  **vs. female**  **HV** |
| --- | --- | --- | --- | --- | --- | --- | --- | --- | --- |
|  | **Male**  **(n=5)** | **Female**  **(n=8)** | **Male**  **(n=5)** | **Female**  **(n=8)** | **Male**  **(n=6)** | **Female**  **(n=14)** | **p** | **p** | **p** |
| **Clinical** |  |  |  |  |  |  |  |  |  |
| HAM-D | 15.00 (5.96) | 17.75 (5.37) | 11.60 (5.60) | 15.00 (6.02) | - | - | .507 | .301 | - |
|  |  |  |  |  |  |  |  |  |  |
| **Biological markers** |  |  |  |  |  |  |  |  |  |
| CRP, mg/L | 3.40 (1.66) | 3.16 (2.61) | 3.46 (1.82) | 4.35 (3.40) | 0.88 (0.49) | 3.17 (4.07) | .435 | .769 | .116 |
| IL-6, pg/mL | 24.22 (37.44) | 7.75 (9.27) | 2.00 (1.58) | 2.25 (1.67) | 1.00 (0.63) | 2.50 (3.52) | .823 | 1.0 | .372 |
| C3, g/L | 1.41 (0.14) | 1.48 (0.25) | 1.43 (0.10) | 1.44 (0.29) | 1.31 (0.14) | 1.25 (0.19) | .724 | 1.0 | .535 |
| C4, g/L | 0.39 (0.08) | 0.31 (0.13) | 0.38 (0.12) | 0.28 (0.12) | 0.27 (0.07) | 0.29 (0.06) | .187 | .213 | .457 |
| Cortisol, mcg/L | 138.00 (32.53) | 91.50 (56.72) | 110.60 (33.37) | 103.38 (35.29) | 98.83 (20.16) | 137.57 (49.74) | .067 | .942 | .137 |
| Leptin, pg/L | 25937.40 (16072.47) | 36811.75 (24062.53) | 26087.60 (20300.72) | 29731.75 (18444.45) | 10006.50 (8254.09) | 21311.08 (16042.69) | .354 | .524 | .029 |
| Adiponectin, ng/L | 6353.00 (2641.38) | 9643.75 (6137.98) | 7006.60 (2379.66) | 8458.86 (4044.50) | 9659.33 (2768.53) | 13707.23 (4343.50) | .284 | .833 | .046 |
| BDNF, pg/mL | 348.02 (126.48) | 701.48 (411.60) | 807.95 (481.01) | 788.70 (570.60) | 621.26 (660.20) | 624.55 (495.34) | .284 | .943 | .968 |

Continuous variables are expressed as mean and standard deviation (SE); vs: versus; HAM-D: Hamilton scale for Depression; CRP: C-Reactive Protein; IL: Interleukin; C3: Complement fraction 3; C4: Complement fraction 4; BDNF: Brain-Derived Neurotrophic Factor.
